# *Toll-Like Receptor 4* Wild Type Homozygozity of Polymorphisms +896 and +1196 Is Associated with High Gastrin Serum Levels and Peptic Ulcer Risk

**DOI:** 10.1371/journal.pone.0131553

**Published:** 2015-07-10

**Authors:** Vesa-Matti Pohjanen, Olli-Pekka Koivurova, Heikki Huhta, Olli Helminen, Johanna M. Mäkinen, Jari M. Karhukorpi, Tapio Joensuu, Pentti O. Koistinen, Jarno M. Valtonen, Seppo E. Niemelä, Riitta A. Karttunen, Tuomo J. Karttunen

**Affiliations:** 1 Medical Research Center Oulu, Oulu University Hospital and University of Oulu, Oulu, Finland; 2 Department of Internal Medicine, Oulu University Hospital, Oulu, Finland; 3 Department of Medical Microbiology and Immunology, Institute of Diagnostics, University of Oulu, Oulu, Finland; 4 Eastern Finland Laboratory Centre Joint Authority Enterprise (ISLAB), Joensuu, Finland; 5 Department of Internal Medicine, Pietarsaari City Hospital, Pietarsaari, Finland; 6 Oulu City Hospital, Oulu, Finland; Centro di Riferimento Oncologico, IRCCS National Cancer Institute, ITALY

## Abstract

Toll-like receptor 4 is a part of the innate immune system and recognizes *Helicobacter pylori* lipopolysaccharide. The goal of this study was to analyze the role of *Toll-like receptor 4* polymorphisms +896 (rs4986790) and +1196 (rs4986791) in the pathogenesis of *Helicobacter pylori* related gastroduodenal diseases in relation to gastric secretion and inflammation. *Toll-like receptor 4 polymorphisms*, serum gastrin-17 and pepsinogen I and II concentrations were determined, and gastroscopies with histopathological analyses were performed to 216 dyspeptic patients. As genotype controls, 179 controls and 61 gastric cancer patients were studied. In our study, the *Toll-like receptor 4* +896 and +1196 polymorphisms were in total linkage disequilibrium. The homozygous wild types displayed higher gastrin-17 serum concentrations than the mutants (p = 0.001) and this effect was independent of *Helicobacter pylori*. The homozygous wild types also displayed an increased risk for peptic ulcers (OR: 4.390). *Toll-like receptor 4* genotypes did not show any association with *Helicobacter pylori* positivity or the features of gastric inflammation. Toll-like receptor 4 expression was seen in gastrin and somatostatin expressing cells of antral mucosa by immunohistochemistry. Our results suggest a role for Toll-like receptor 4 in gastric acid regulation and that the *Toll-like receptor 4* +896 and +1196 wild type homozygozity increases peptic ulcer risk via gastrin secretion.

## Introduction

Toll-like receptor 4 (TLR4) is a part of a pattern recognition molecule family. TLR4 binds several microbial ligands and the subsequent downstream signaling stimulates cytokine production creating a proinflammatory environment. The bacterial lipopolysaccharides (LPS) of *Helicobacter pylori* and other gram negative bacteria are TLR4 target molecules and thus TLR4 has been thought to have a role in *H*. *pylori* related diseases [1,2].

The gene encoding TLR4 in humans is located on chromosome locus 9q32-q33 and contains 4 exons. Two non-synonymous polymorphisms *TLR4* +896 adenine/guanine (rs4986790) and *TLR4* +1196 cytosine/thymine (rs4986791) have been located in the fourth exon causing amino acid substitutions: glycine for aspartic acid at 299 position and isoleucine for threonine at 399 position respectively [2]. These two polymorphisms are in linkage disequilibrium, and 6 –14% of Indo-European individuals are double heterozygotes for these alleles [3]. Both of these mutations are associated with LPS hyporesponsiveness and the double mutant even more prominently so [2].

Previous reports have shown contradictory associations between the *TLR4* +896 and +1196 polymorphisms and *H*. *pylori* related gastritis and peptic ulcer and have not presented a clear physiological mechanism for the polymorphisms in their pathogenesis [4–7]. The key mechanism in the pathogenesis of most peptic ulcers is thought to be *H*. *pylori* induced excessive gastrin secretion and the following excessive acid secretion, but the mechanisms of such aberration of regulation are unclear [8,9]. However, physiological connections between TLR4 and gastrin secretion has been documented in animal models [10,11] but there are no studies about the possible role of *TLR4* polymorphisms in gastrin secretion in humans.

We hypothesized that TLR4 could affect gastrin levels and thereby affect peptic ulcer pathogenesis also in human subjects. We have investigated the relation between the *TLR4* +896 and +1196 polymorphisms, and serum gastrin-17 (G17) and pepsinogen I (PGI) and II (PGII) concentrations, *H*. *pylori* infection and histopathologic gastric inflammation in dyspeptic patients. We also compared the genotype distribution between peptic ulcer, non-ulcer dyspepsia, gastric cancer and control patients.

## Patients and Methods

The patient group comprised of 216 dyspeptic patients collected from three hospitals in the city of Oulu, Finland, all performing outpatient endoscopies. Exclusion criteria were following: treated *H*. *pylori* infection, treatment with immunosuppressive drugs, ongoing antibiotic treatment and previous gastric surgery. The patients were inquired for the use of antacids, sucralfate, histamine 2 receptor antagonists or proton pump inhibitors. Upper gastrointestinal endoscopies were performed by experienced endoscopists. Endoscopy findings, including the presence or absence of gastric or duodenal ulcer, were registered. Biopsies form the descending part of duodenum, gastric antrum and gastric body were taken for histological analysis [12].

A series of 61 patients with gastric cancer was collected during the years 1996–2000 in Oulu University Hospital and represents an unselected series of patients treated by surgery. The control group consisted of university staff and students of which no data were collected concerning dyspeptic symptoms or visits to gastroenterologists. All controls and study subjects originated from the homogenic ethnically Finnish population. The groups have been described previously [13,14].

The control and dyspeptic patients’ DNA was extracted from blood leucocytes and, in patients with gastric cancer, from a fresh frozen gastric tissue specimen representing non-neoplastic tissue. Extraction was performed as previously described [13]. PCR tests from the DNA samples were performed to detect the *TLR4* +896 and +1196 polymorphisms as previously described [15]. The investigators who performed the genetic analyses were blinded to the clinical data, and the clinical investigators were blinded to the genetic data.

*H*. *pylori* status was analyzed in the dyspeptic patients. Positive *H*. *pylori* status was based on a positive serology and a positive bacterial culture or a PCR test as previously described [13]. The presence of the pathogenetic *H*. *pylori* gene variant, *cagA*, was detected as previously described [16]. G17, PGI and PGII measurements were performed with GastroPanel assays by Biohit (Helsinki, Finland) laboratory from the patients’ serum samples.

For immunohistochemical stainings, formalin fixed and paraffin embedded sections were pretreated by heating with microwaves in ethylenediaminetetraacetic acid (for single stains) or sodium citrate (pH 6, for double stains) with 850W for 10 minutes for antigen retrieval. The immunohistochemical stainings were performed with mouse monoclonal antibody against human TLR4 (1:1000, H00007099-M02, Abnova, Taipei, Taiwan), rabbit polyclonal antibody against human gastrin-17 (1:250, A 0568, Dako, Glostrup, Denmark) and rabbit polyclonal antibody against human somatostatin (1:600, A0566, Dako). The incubation time was 60 minutes for TLR4 and somatostatin antibodies and 30 minutes for gastrin antibody at room temperature. For the detection of the antibody reaction we used EnVision detection kit and the double stains of TLR4, gastrin and somatostatin were performed by EnVision G|2 Doublestain kit (Dako) with related protocols. The validation of immunohistochemical analysis was performed with negative controls including buffer solution or irrelevant antibodies instead of gastrin and TLR4 antibodies. Duodenal epithelial cells were used as a positive control for TLR4 [17]. Expression intensity of TLR4 in different cell populations of gastric mucosa was assessed by using single stained sections and a scale from negative to weak, moderate and strong expression.

In the dyspeptic patients’ group, 129 patients had no signs of active or inactive peptic ulcer and had no previously diagnosed ulcer. In the group, 50 dyspeptic patients had an active duodenal ulcer seen in the endoscopy, 23 patients had an active gastric ulcer, 5 patients had a previously diagnosed duodenal ulcer or duodenal scarring consistent with a duodenal ulcer and 9 patients had a previously diagnosed gastric ulcer or scarring in the stomach consistent with a gastric ulcer. In the ulcer risk tests with the control group, where we used only permanent cofactors, we included all the previously diagnosed patients into the ulcer group. When we did ulcer risk analyses between the ulcer and non-ulcer patients’ group and also used non-permanent risk factors as *H*. *pylori* positivity and smoking, we excluded all the patients with inactive ulcers from the analysis to prevent them from confounding the results.

The gastric cancers were re-classified by two pathologists (JMM and TJK). Other histopathologic changes in the gastric mucosa were analyzed according to the Sydney system [18] by one pathologist (TJK) blinded for clinical and genetic data. The amount of duodenal lamina propria mononuclear cells was evaluated by using a four point scale (0–3) as described previously [12].

Medians and interquartile ranges (IQR) are reported for skewed continuous variables (G17, PGI and PGII). The effects of the risk factors on the serum levels of G17, PGI and PGII were analyzed with Mann-Whitney U test and binary logistic regression models. The risks of diseases associated with the genetic polymorphisms were analyzed with binary logistic regression models. Forward likelihood ratio criteria were used in stepwise multifactorial regression models. The correlations between the genetic polymorphisms and the histological variables were analyzed with Mann-Whitney U test. P-value of less than 0.05 was considered statistically significant. Odds ratios (ORs) and the corresponding 95% confidence intervals (CIs) were calculated from the regression models. Missing data were excluded pairwise from the analyses. The data were analyzed using the SPSS software version 19 (IBM, Armonk, New York, United States).

The study followed the guidelines of the declaration of Helsinki and was approved by the regional ethics committee of the Northern Ostrobothnia Hospital District. Dyspeptic patients gave written informed consent and control patients gave verbal informed consent. The cancer patient data and tissue samples were obtain from the archives of Oulu University Hospital. A permission to use the data after de-identification procedure by a third party was obtained from the ethics committee.

## Results

In all of our study populations, the *TLR4* +896 and +1196 polymorphisms were in total linkage disequilibrium so all subjects who had the mutant allele for *TLR4* +896 had also the mutant allele for *TLR4* +1196 and vice versa. The genotype frequencies in the control and non-ulcer dyspepsia groups are consistent with the Hardy-Weinberg equilibrium. Subject group characteristics and genotype frequencies are displayed in Table 1. For the analyses, the *TLR4* +896/+1196 heterozygous and homozygous double mutants were combined into a single group. *H*. *pylori* positivity did not associate with the *TLR4* genotypes in the ulcer or non-ulcer dyspepsia patients’ groups or in the combined group; 62.1% (110/177) of the *TLR4* homozygous wild type patients and 51.3% (20/39) of *TLR4* heterozygous or homozygous mutant patients were *H*. *pylori* positive (p = 0.210).

**Table 1:** Characteristics of the subject groups.

	Subject group				
	Control	Non-ulcer dyspepsia	Active peptic ulcer	Inactive peptic ulcer	Gastric cancer
N	179	129	73	14	61
Male	56 (31.3%)	48 (37.2%)	34 (46.6%)	7 (50.0%)	31 (50.8%)
Female	123 (68.7%)	81 (62.8%)	39 (53.4%)	7 (50.0%)	30 (49.2%)
Mean age (SD^1^)	39.3 (13.4)	52.0 (14.0)	56.6 (13.6)	55.2 (16.0)	65.9 (12.6)
*H*. *pylori* positive	No data	51/129 (39.5%)	71/73 (97.3%)	8/14 (57.1%)	No data
*cagA* positive	No data	39/47 (83.0%)	50/52 (96.2%)	8/8 (100%)	No data
Smoking	No data	21/129 (16.3%)	29/73 (39.7%)	3/14 (21.4%)	No data
On medication^2^	No data	21/129 (16.3%)	33/73 (45.2%)	4/13 (30.8%)	No data
*TLR4* wild type	143 (79.9%)	100 (77.5%)	65 (89.0%)	12 (85.7%)	47 (77.0%)
*TLR4* heterozygous mutant	35 (19.6%)	28 (21.7%)	7 (9.6%)	2 (14.3%)	12 (19.7%)
*TLR4* homozygous mutant	1 (0.6%)	1 (0.8%)	1 (1.4%)	0 (0.0%)	2 (3.3%)

^1^Standard deviation

^2^Proton pump inhibitor, histamine receptor 2 antagonist, sucralfate or antacid

**Fig 1:**
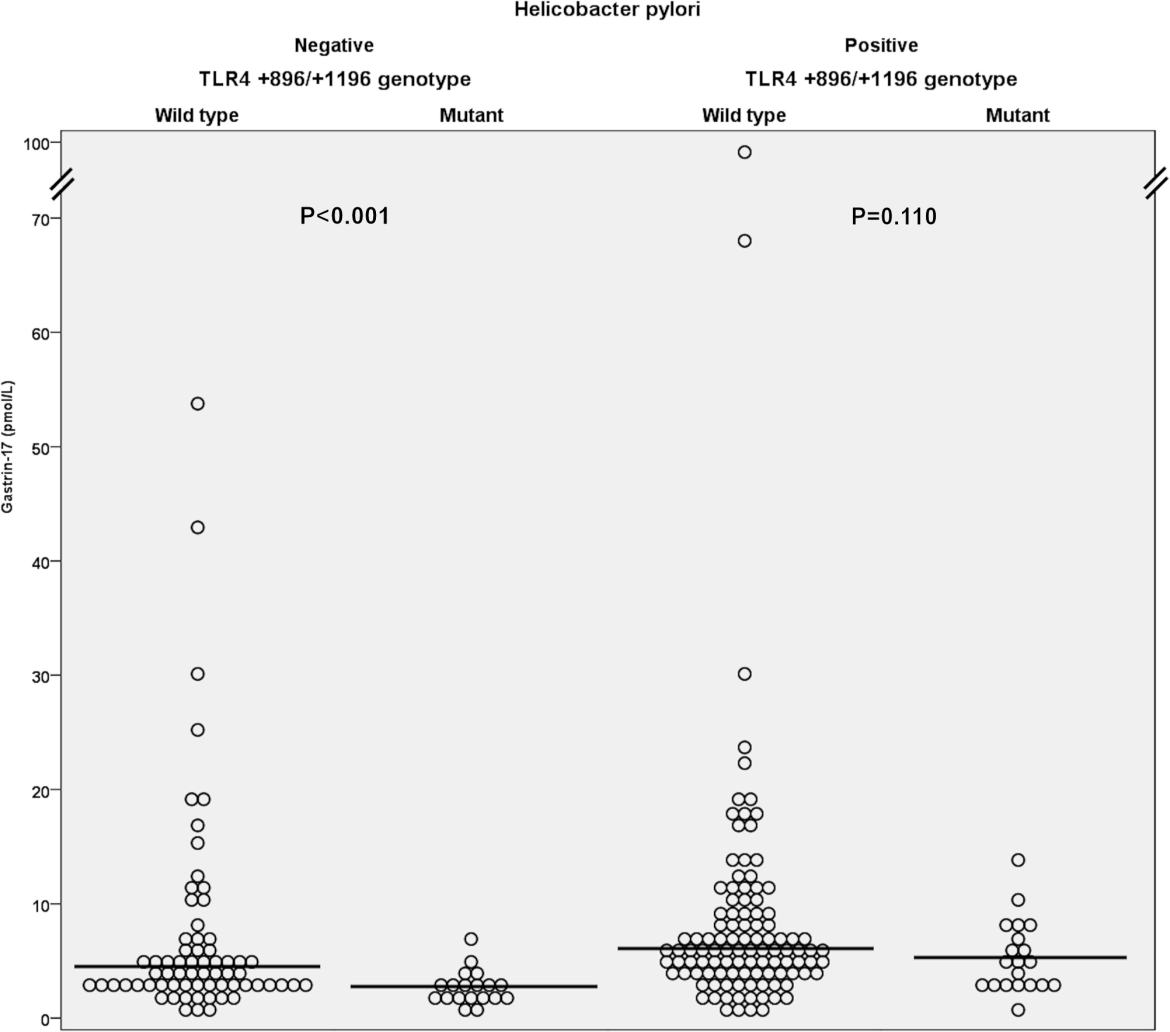
Dot diagram of gastrin-17 serum level distributions in the *TLR4* +896/+1196 genotypes with median value lines. P-values are calculated with Mann-Whitney U test.

Serum G17 serum levels were higher in the *TLR4* wild type patients (median 5.0 pmol/l; IQR: 5.3 pmol/l; n = 174) compared to the heterozygous and homozygous mutants (median 3.1 pmol/l; IQR: 3.2 pmol/l; n = 39; p = 0.001). This difference was also evident in the *H*. *pylori* negative subgroup (respectively, median 4.1 pmol/l; IQR: 3.7 pmol/l; n = 67 and median 2.4 pmol/l; IQR: 1.8 pmol/l; n = 19; p = 0.001), and a similar trend was seen in the *H*. *pylori* positive patients (Fig 1). The *TLR4* homozygous wild types had also higher PGI (respectively, median 93.1 μg/l; IQR: 43.3 μg/l; n = 171 and median 80.2 μg/l; IQR: 45.8 μg/l; n = 39; p = 0.035) and PGII (respectively, median 11.2 μg/l; IQR: 9.8 μg/l; n = 177 and median 7.8 μg/l; IQR: 7.8 μg/l; n = 39; p = 0.005) serum levels compared to the heterozygous and homozygous mutants.

As expected [8], serum G17 concentrations were higher in the *H*. *pylori* positive patients than in the negative ones (respectively, median 5.7 pmol/l; IQR: 5.2 pmol/l; n = 127 and median 3.3 pmol/l; IQR: 3.0 pmol/l; n = 86; p = 0.001). The use of proton pump inhibitor medication (n = 15) or other dyspepsia medication (n = 43) did not affect G17 serum levels significantly. To take account of the effects of multiple variables on the G17 levels, we performed a stepwise logistic regression model. In the model, the *TLR4* wild type genotypes were more common in the patients who surpassed the upper reference limit for serum gastrin concentration provided by the test manufacturer (Biohit) of 7.0 pmol/l (27.2%; 58/213) with an OR of 2.835 (CI: 1.040–7.728; p = 0.042). This model also recognized *H*. *pylori* positivity as a risk factor for high gastrin levels (OR: 2.297; CI: 1.170–4.510; p = 0.016), but indicated the absence of role for age, use of medication, smoking and sex.

To assess if the observed effect of *TLR4* +896 and +1196 polymorphisms on G17 levels affect the incidence of peptic ulcer and gastric cancer, we compared the genotype distributions between the subject groups. The ulcer group including both active and inactive ulcers was compared to the control group by a binary logistic regression analysis: the homozygous wild types had a significant association with peptic ulcers (OR: 2.423; CI: 1.022–5.746; p = 0.045) against heterozygous and homozygous mutants with age (per year OR: 1.098; CI: 1.070–1.126; p<0.001) and sex (male OR: 1.910; CI: 1.028–3.550; p = 0.041) as covariates. The duodenal and gastric ulcer groups displayed similar genotype distributions compared to each other but the separate tests were not statistically significant obviously due to low amount of patients. The *TLR4* genotype distribution of the gastric cancer group did not differ significantly from the control subjects’ genotype distribution in a similar model.

To take the *H*. *pylori* and *cagA* status into account, we compared the active ulcer group to the non-ulcer dyspepsia group in crude and adjusted logistic regression models. In the crude model, the homozygous wild types displayed an increased risk for ulcers with an OR of 2.356 (Table 2). In the stepwise models, we used *TLR4* polymorphisms, *H*. *pylori* or *cagA* positivity, sex, age and smoking as covariates. The first multifactorial model with *H*. *pylori* positivity as a cofactor recognized only *H*. *pylori* (OR: 55.79; CI: 12.84–242.3; p<0.001) and smoking (OR: 3.614; CI: 1.535–0.508; p = 0.003) as risk factors, which was unexpected based on the other results. However, on the second model, where we accounted for *cagA* positivity, we saw a statistically significant risk increase associated also with the *TLR4* homozygous wild types (OR: 4.390).

**Table 2:** Peptic ulcer risk.

	Crude model			Adjusted model^4^		
	P-value	OR^2^	CI^3^	P-value	OR	CI
*TLR4* homozygous wild type^1^	0.046	2.356	1.014–5.473	0.032	4.390	1.134–16.998
*H*. *pylori* positivity	<0.001	54.294	12.749–231.215	-	-	-
*cagA* positivity	0.046	5.128	1.030–25.529	0.037	6.221	1.117–34.644
Smoking	<0.001	3.390	1.748–6.571	0.001	5.491	1.959–15.388
Age, per year	0.028	1.024	1.003–1.046	-	-	-
Male	0.194	1.471	0.822–2.633	-	-	-

Active peptic ulcer risk in comparison with the non-ulcer dyspepsia patients in logistic regression models.

^1^Versus +896/+1196 heterozygous and homozygous mutants

^2^Odds ratio

^3^95% confidence interval

^4^Stepwise forward (likelihood ratio criteria) model with *TLR4* polymorphisms, *cagA* positivity, smoking, age and sex as covariates.

We also analyzed the association of the *TLR4* polymorphisms and the histological findings of the dyspepsia and ulcer patients’ gastric and duodenal biopsies. The *TLR4* polymorphisms did not correlate with the Sydney system based variables of chronic or active gastritis, atrophy, intestinal metaplasia or *H*. *pylori* score (Table 3). The only difference was that the *TLR4* homozygous wild types displayed slightly higher scores of duodenal lymphocytes (mean score 1.04 versus 0.92; p = 0.013).

**Table 3:** *TLR4* genotypes and gastric histology.

	*Helicobacter pylori* negative			*Helicobacter pylori* positive		
*TLR4* +896/+1196 genotype	Wild type	Mutant^1^	P-value^2^	Wild type	Mutant	P-value
Antrum mononuclear cells (0–3; n = 193)	0.19	0.17	0.884	2.32	2.30	0.797
Antrum neutrophils (0–3; n = 212)	0.04	0.00	0.604	1.47	1.45	0.952
Antrum atrophy (0–3; n = 209)	0.05	0.00	0.373	0.95	1.25	0.162
Body mononuclear cells (0–3; n = 204)	0.21	0.16	0.706	1.89	1.89	0.864
Body neutrophils (0–3; n = 207)	0.00	0.00	1.000	0.66	0.78	0.626
Body atrophy (0–3; n = 207)	0.09	0.00	0.449	0.17	0.11	0.648

Variables are based on the Sydney system. Both non-ulcer dyspepsia and ulcer patients were included in analysis. Mean values are indicated.

^1^Heterozygotes and homozygotes

^2^Mann-Whitney U test

**Fig 2:**
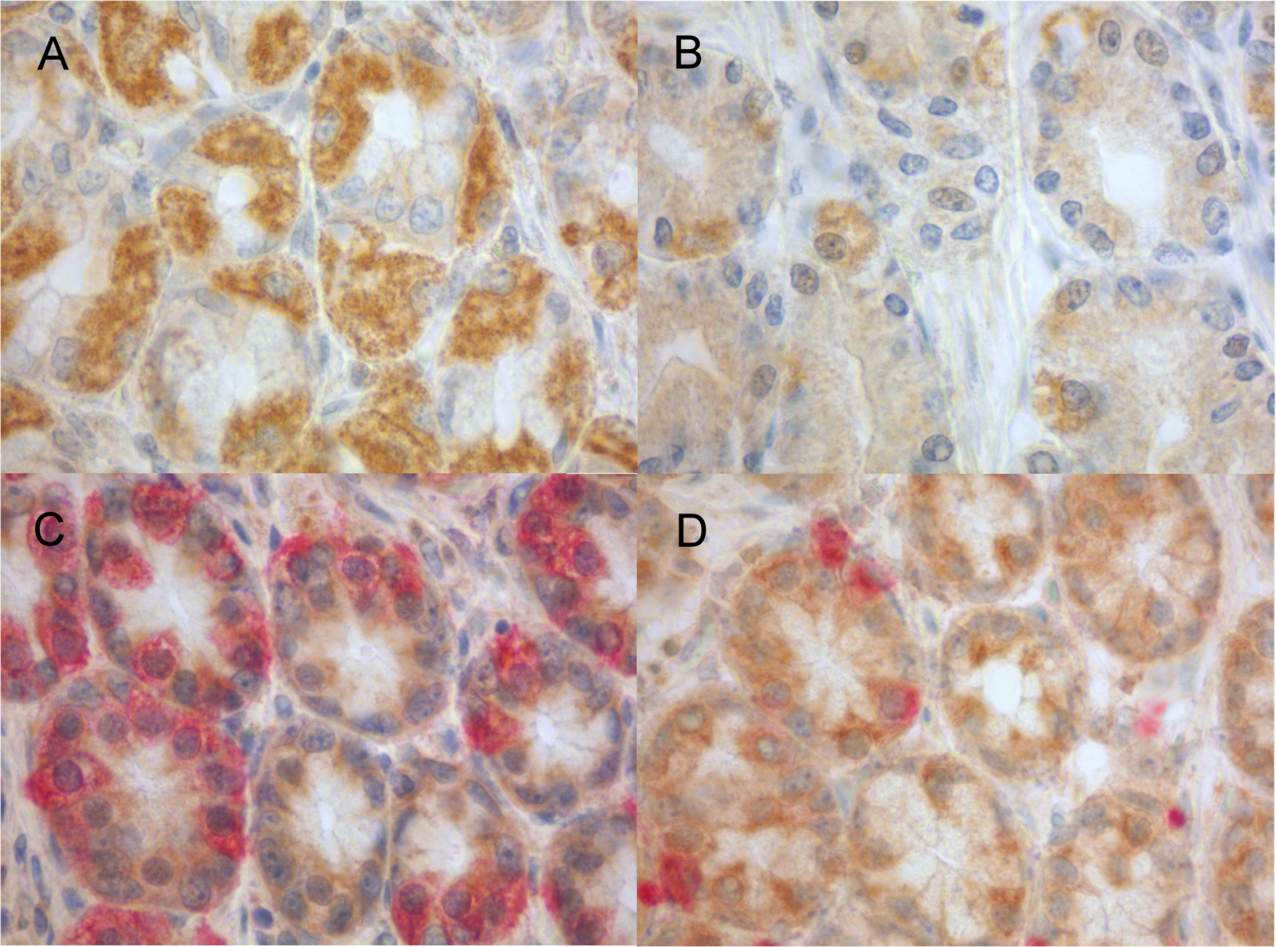
Microphotographs demonstrating TLR4 expression in gastric mucosa. The immunohistochemical staining of TLR4 expression in the body glands show expression mainly in the parietal cells (A), while in the glandular neck zone of the antrum (B) of stomach only occasional cells are positive. Double stainings (C, D) show TLR4 positivity (brown) in gastrin positive cells (red, C) and in somatostatin positive cells (red, D) in the antrum.

To assess the expression of TLR4 in relation to gastrin secreting G cells and somatostatin secreting D cells in the antral mucosa and expression patterns in the body mucosa, we used immunohistochemical single and double stainings for samples representing normal human antral and body mucosa (Fig 2). Strong TLR4 expression was seen in the cytoplasm of epithelial cells in gastric surface and the upper parts of the foveolar epithelium. In the antrum glandular neck zone, the epithelial cells were positive with weak to moderate expression levels. However, some cells were moderately to strongly positive and some cells were weakly stained or negative as were majority of cells in the antral glands. The double stained slides showed that majority of the gastrin expressing cells present in the glandular neck were TLR4 positive, and that the majority of somatostatin expressing cells were similarly TLR4 positive. These expression patterns suggest that the majority of moderately or strongly TLR4 positive cells in the antral glandular neck region are G cells and D cells. In the body glands TLR4 immunopositivity was present in the parietal cells, where the expression varied from mild to moderate but some individual cells were strongly positive.

## Discussion

To our knowledge, our study is the first one to document that the *TLR4* +896/+1196 polymorphisms affect gastrin levels. The *TLR4* homozygous wild type patients had higher G17 serum levels and had more reference limit surpassing scores in the multifactorial model. As an indirect evidence of possible physiological link between TLR4 and gastric secretion, we demonstrate TLR4 expression in the gastrin and somatostatin expressing cells of the antral mucosa. Additionally, we report an increased risk for peptic ulcer for the *TLR4* +896/+1196 homozygous wild type patients over the double mutant polymorphism carriers, which is physiologically appropriate considering the G17 results. Since the ulcer risk genotype of *TLR4* associated with high serum gastrin but not with gastric inflammation, we suggest that the role of TLR4 in the regulation of gastric secretion is more important in mediating the ulcer risk than its direct proinflammatory effects.

Hypergastrinemia and increased gastric acid output are well known abnormalities associated especially to the duodenal ulcer phenotype of *H*. *pylori* related gastroduodenal disease, but the specific mechanisms of *H*. *pylori* related hypergastrinemia are not known [8,9]. Animal models have previously documented a connection between activation of innate immunity and increased gastrin secretion [10]. Interestingly, murine G cells express TLR4, and in vitro experiments have shown that LPS, a major ligand of TLR4, induces secretion of gastrin from G cells presumably by TLR4 mediated mechanisms [11]. Somatostatin is the primary inhibitor of gastrin stimulated acid secretion with effects on the gastrin secretion of G cells and directly on parietal cells. In physiological conditions the secretion of somatostatin by D cells is induced by the acidity sensed by the cells. Gastric infection has been associated with the suppression of somatostatin secretion suggesting that either direct or indirect recognition of bacterial products is important in the regulation of D cells [19].

Our immunohistochemical studies of gastric antral mucosa indicate, that in addition to the expression in the surface and foveolar epithelium, TLR4 positivity is seen in the glandular neck zone and it is located predominantly in the gastrin and somatostatin expressing cells, *i*.*e*. G and D cells. In humans, the presence of TLR4 in G and D cells has not been previously reported. Schmausser *et al*. detected TLR4 in human foveolar epithelium but did not comment on G or D cells [20]. We speculate that TLR4–ligand interaction in G and D cells could have a direct effect in the gastrin and somatostatin secretion, respectively, and potentially explain the associations between the *TLR4* polymorphisms and increased serum gastrin and the peptic ulcer risk. However, evaluation of the physiological role of TLR4 in human G and D cells needs additional studies. We observed also heterogeneous expression of TLR4 in parietal cells in the body mucosa. This has not been previously documented, and the physiological role of TLR4 expression in parietal cells needs further studies. However, TLR4 in parietal cells could also be involved in the innate immune defense by gastric acid regulation.

Physiologically, enhanced gastrin secretion in response to TLR4 activation can be considered appropriate as TLR4 is essential for the innate immunity against gram negative bacteria and gastric acid secretion is a part of the immunological surveillance mechanism in the gastrointestinal tract. The *TLR4* +896 and +1196 wild type receptors have been documented to be more responsive to LPS than the mutant receptors [2]. So in cases where *H*. *pylori* is able to avoid or locally neutralize the low pH, the constant activation of wild type *TLR4* +896/+1196 receptors could be hypothesized to lead to hypergastrinemia and increased acid load leading to an ulcer.

In our study, the higher gastrin levels in the *TLR4* homozygous wild types could not be explained by atrophic gastritis as no association between atrophic gastritis and *TLR4* genotypes was found. Also the PGI and PGII levels were similarly higher in the *TLR4* homozygous wild types indicating functional secretion in relation to the increased gastrin levels both in the body and antrum of the stomach. According to our multivariate analyses, the G17 increase was only explained by the differences in the *TLR4* genotypes and *H*. *pylori* positivity and, for example, not by the use of acid secretion affecting drugs including proton pump inhibitors.

The *TLR4* +896/+1196 homozygous wild types and high G17 levels were linked also in the *H*. *pylori* negative subjects. This could result from TLR4 recognizing the LPS of other gram negative microbial flora present within the gastric contents. Interestingly, the Kidd *et al*. murine G cell model showed higher gastrin secretion stimulating potency for *Salmonella enteritidis* and *Escherichia coli* LPS than for *H*. *pylori* LPS [11].

*TLR4* polymorphisms have been proposed to affect the probability of disease either through increased susceptibility for bacterial infections or through altered inflammatory states [1,2]. Defective TLR4 function has been suggested to lead to a pro-inflammatory response due to a deficiency in the anti-inflammatory interleukin 10 [1]. Previous studies on *TLR4* polymorphisms and gastric inflammation have provided conflicting results: Achuyt *et al*. reported higher neutrophil scores for *TLR4* +896 mutants and higher plasma cell scores for +1196 mutants [4]. Rigoli *et al*. associated +896 mutants to both antrum and body predominant gastritis and +1196 mutants to body predominant gastritis [6]. Bagheri *et al*. reported higher mononuclear cell scores for +896 mutants, no significant difference in polymorphonuclear cell scores, but still the wild types were more prevalent in active chronic gastritis group when compared to the chronic gastritis group [7]. There are also several studies where no associations between these *TLR4* polymorphisms and gastric inflammation were seen [21–23]. In our study, we saw no difference in the prevalence of *H*. *pylori* infection or Sydney system based histological inflammatory markers between the *TLR4* genotypes. The *TLR4* homozygous wild types were only associated with a slightly increased lymphocyte count in duodenum, which could also be a secondary effect, related with the increased gastric acid secretion. However, since specimens of the actual ulcers were not studied, we cannot exclude the possibility of pro-inflammatory effects manifesting within or in the vicinity the ulcer.

There is only one study reporting an association between these *TLR4* polymorphisms and peptic ulcer. The *TLR4* +896 mutants were associated with duodenal ulcers in a study, which, however, had technical problems, as in over 40% of the duodenal ulcer patients the *TLR4* +896 polymorphism could not be analyzed [5]. In our study, the risk of peptic ulcer was increased in the *TLR4* homozygous wild types and it persisted also after accounting for other risk factors with multivariate analyses. Our ulcer group was heterogeneous in respect of the location of the ulcers and the significance of non-steroidal anti-inflammatory drug (NSAID) use cannot be excluded. However, it should be noted that in addition of having a key role in *H*. *pylori* related ulcers, gastric acid secretion also has a major role in NSAID related ulcers [24]. Thus TLR4 activation and its effects on gastrin and acid secretion might also have a role in the pathogenesis of NSAID ulcers.

We did not observe associations between the *TLR4* genotypes and gastric cancer. *TLR4* +896 and +1196 mutant polymorphisms have both been associated with gastric cancer risk in a meta-analysis [25]. The lack of association in our study might be due to the small amount of subjects or the composition of the cancer group.

In conclusion, we document here that the *TLR4* +896/+1196 homozygous wild types have increased G17 serum levels. We also show that antral G and D cells express TLR4 suggesting a potential physiological link between TLR4 and the regulation of gastrin secretion. In line with the association with increased gastrin levels, we show that the *TLR4* +896/+1196 homozygous wild types have an increased risk for peptic ulcers over the double mutant +896/+1196 allele carriers and the ulcer risk association was seen against the control group and the non-ulcer dyspepsia group. We suggest that the effects of *TLR4* polymorphisms on gastrin could lead to the ulcer risk by enhanced TLR4 activation in G and D cells and the potentially following activation on gastric acid secretion. Confirmation of this pathophysiological link needs additional studies, but might provide possibilities for new treatment strategies in gastric acid related diseases, as many compounds able to activate or inhibit TLR4 have been recently recognized [26].

## Supporting Information

S1 Dataset Patient data used in the analyses. (XLSX)
